# Chimeric Antigen Receptor T-Cell Therapy: What We Expect Soon

**DOI:** 10.3390/ijms232113332

**Published:** 2022-11-01

**Authors:** Massimo Martino, Virginia Naso, Barbara Loteta, Filippo Antonio Canale, Marta Pugliese, Caterina Alati, Gerardo Musuraca, Davide Nappi, Anna Gaimari, Fabio Nicolini, Massimiliano Mazza, Sara Bravaccini, Daniele Derudas, Giovanni Martinelli, Claudio Cerchione

**Affiliations:** 1 Stem Cell Transplant and Cellular Therapies Unit, Great Metropolitan Hospital “Bianchi-Melacrino-Morelli”, 89133 Reggio Calabria, Italy; 2 Stem Cell Transplant Program CIC 587, Great Metropolitan Hospital “Bianchi-Melacrino-Morelli”, 89133 Reggio Calabria, Italy; 3 Hematology Unit, Great Metropolitan Hospital “Bianchi-Melacrino-Morelli”, 89133 Reggio Calabria, Italy; 4 Hematology Unit, IRCCS Istituto Romagnolo per lo Studio dei Tumori (IRST) “Dino Amadori”, 47014 Meldola, Italy; 5 Department of Hematology and Cell Bone Marrow Transplantation (CBMT), Ospedale di Bolzano, 39100 Bolzano, Italy; 6 Immunotherapy, Cell Therapy and Biobank (ITCB), IRCCS Istituto Romagnolo per lo Studio dei Tumori (IRST) “Dino Amadori”, 47014 Meldola, Italy; 7 Biosciences Laboratory, IRCCS Istituto Romagnolo per lo Studio dei Tumori (IRST) “Dino Amadori”, 47014 Meldola, Italy; 8 S.C. di Ematologia e C.T.M.O., Ospedale Oncologico di Riferimento Regionale “A. Businco”, 09121 Cagliari, Italy; 9 Scientific Directorate IRCCS Istituto Romagnolo per lo Studio dei Tumori (IRST) “Dino Amadori”, 47014 Meldola, Italy

**Keywords:** CAR-T, manufacturing, toxicities, solid tumor, DRG, cost

## Abstract

The treatment landscape for hematologic malignancies has changed since the recent approval of highly effective chimeric antigen receptor T-cell therapies (CAR-T). Moreover, more than 600 active trials are currently ongoing. However, early enthusiasm should be tempered since several issues are still unsolved and represent the challenges for the coming years. The lack of initial responses and early relapse are some hurdles to be tackled. Moreover, new strategies are needed to increase the safety profile or shorten the manufacturing process during CAR-T cells therapy production. Nowadays, most clinically evaluated CAR-T cells products are derived from autologous immune cells. The use of allogeneic CAR-T cells products generated using cells from healthy donors has the potential to change the scenario and overcome many of these limitations. In addition, CAR-T cells carry a high price tag, and there is an urgent need to understand how to pay for these therapies as many of today’s current payment systems do not feature the functionality to address the reimbursement gap. Finally, the clinical experience with CAR-T cells for solid tumors has been less encouraging, and development in this setting is desirable.

## 1. Introduction

Chimeric antigen receptor T-cell therapy (CAR-T) is a type of immunotherapy in which a patient’s T cells, immune cells with anti-cancer activity, are collected and genetically engineered to improve their tropism and promote the elimination of cancer cells [1,2,3,4]. The modified cells are expanded in the laboratory and then returned to the patient to fight cancer. The year 2018 represents a milestone in the history of medicine: the Food and Drug Administration (FDA) [5,6] and the European Medicines Agency (EMA) [7] approved the first two products containing autologous T cells genetically modified ex vivo, tisagenlecleucel and axicabtageneciloleucel, for commercial use, revolutionizing the treatment landscape for relapsed or refractory (R/R) ALL and R/R non-Hodgkin lymphoma (NHL). However, history goes on, and in the last year, the FDA approved lisocabtagenemaraleucel in R/R NHL [8] and idecabtagenevicleucel in R/R multiple myeloma (MM) [9,10,11]. In addition, brexucabtageneautoleucel has been approved for treating adult patients with R/R mantle cell lymphoma [12] and is now being studied in patients with R/R B-ALL [13]. Moreover, the rolling submission of a biologics license application has been completed to support the approval of the investigational ciltacabtageneautoleucel in R/R MM [14]. Researchers also published data about an autologous CAR-T that uses a novel binding domain to target a B-cell maturation antigen (CARTddBCMA) in R/R MM, designed to reduce the risk of immunogenicity, while increasing stability [15]. The main characteristics of the constructs and the clinical indications are summarized in Table 1.

The approval by the FDA and EMA and the available therapies mentioned above have significantly impacted the treatment of several hematologic malignancies. Moreover, out of the 600 active trials, ongoing FDA and EMA estimate to approve 10–20 gene therapy products per year by 2025. The growth of CAR-T cells therapies mirrors the extraordinary results of the pivotal studies. In lymphoid malignancies, the overall response rates were 70% to 80%, with complete responses observed in approximately 50% of patients [16,17,18,19,20]. The BCMA–CAR-T is associated with a median progression-free survival of 8.8 months, more significant than expected from other available therapies in heavily pretreated R/R MM [21].

Despite these unprecedented gains, numerous issues must be solved, representing the goal for the coming years. The significant challenges are the lack of initial responses and early relapse, driven by antigen escape and T-cell exhaustion. Developing strategies to increase the safety profile is mandatory to maintain the integrity of CAR-T cells therapy product manufacturing, resulting in longer gaps in treatment in patients who require urgent disease control. Novel approaches to shorten the manufacturing process are developing. Most clinically evaluated CAR-T cells products have been derived from autologous immune cells. The use of allogeneic CAR-T cells products generated using cells from healthy donors has the potential to overcome many of these limitations. The clinical experience with CAR-T cells therapy for solid tumors has been less encouraging, and development in this setting is desirable. Finally, CAR-T cells carries a high price tag, and there is an urgent need to understand how to pay for these therapies.

**Figure 1:**
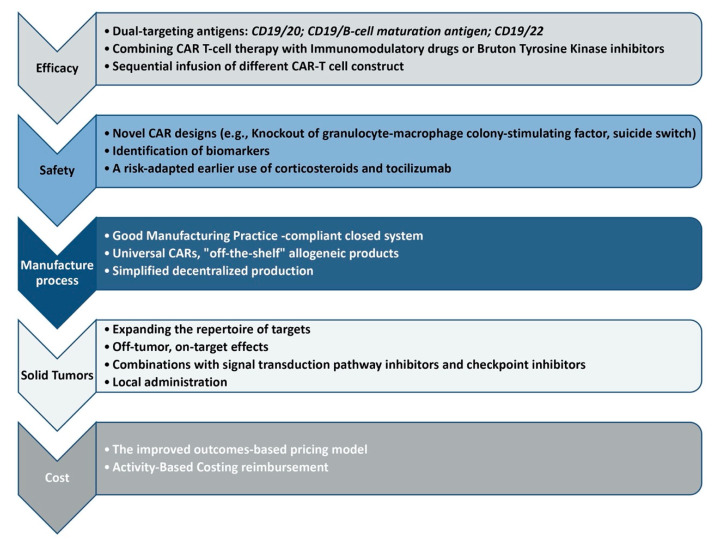
Future directions for chimeric Antigen Receptor Cell Therapy (CAR-T).

The purpose of this paper is to provide a succinct summary of the CAR-T cells problems to be solved in the future (Figure 1).

## 2. Improving Responses

With the longer follow-up reported, we are closer to answering the question of whether sustained remissions are possible with CAR-T cells monotherapy. Unfortunately, despite initially impressive in-depth responses, more than half of the patients experienced a relapse.

The failure is primarily mediated by antigen escape and T-cell exhaustion. In total, 33% of the relapses in ZUMA-1 were CD19 negative [22]. Similarly, the loss of BCMA expression contributes to disease relapse in MM [23].

One strategy to improve efficacy is finding novel targets and designing constructs targeting more than one antigen, such as the dual-targeting CD19/CD22 CAR in B-cell malignancies [24,25,26], dual CD19/CD20 CAR in lymphomas and leukemia [27,28], and CD19/BCMA-specific CAR in MM [29].

CD22-directed CAR-T cells have shown efficacy against leukemia [30] and lymphoma [31], representing the first alternative CAR target with comparable efficacy to CD19 CAR-T.

Dual-targeting CARs or combination therapy may prevent relapses due to escape variants; therefore, they are the way forward.

Other studies investigate the sequential infusion of CD19- CAR-T and BCMA-CAR-Ts for R/R MM [29]. The preliminary results evidence the tolerability and efficacy of this approach, and present a simple and safe design applicable for the establishment of multiple CAR-T cells therapies. In addition, de Larrea and colleagues evaluated strategies for simultaneously targeting BCMA and G-protein–coupled receptor class C group 5D (GPRC5D) with CAR-T cells in MM. Their study demonstrates that it was feasible to simultaneously target GPRC5D and BCMA, and provides insight into optimal dual targeting designs; thus, broadening the arsenal and potential efficacy of cellular therapies for MM. Another strategy is combining CAR-T cells with small molecules to increase surface expression of the target, as in the clinical trial combining BCMA CAR-T with a gamma-secretase inhibitor, which blocks BCMA cleavage [32].

It is now recognized that T-cell phenotype and fitness correlate with clinical response. Several predictive biomarkers of T-cell function (such as LAG3, TIM3, and PD-1 expression) are being identified [33]. CAR-T cells products with lower naive, central memory phenotype in lymphoma show worse T-cell responses [34]; similarly, in myeloma, a lower CD4:CD8 ratio and higher terminally exhausted T cells in non-responders [35]. Several strategies are being pursued to optimize the CAR-T cells product composition, including balancing T-cell subset ratios [19], combining CAR-T cells with lenalidomide [36,37] or Bruton tyrosine kinase (BTK) inhibitors [38]. The administration of lenalidomide, in combination with anti-BCMA CAR-T, may be warranted based on tumoricidal effects, a more permissive tumor microenvironment for CAR-T function, and the observed intrinsic impact on CAR-T function. Qin et al. postulated that dosing with a BTK inhibitor before CAR-T cells engraftment could reduce the tumor size, and normalize immune functions and microenvironment conditions. Concurrent dosing with a BTK inhibitor after administration of CD19-targeted CAR-T therapy may mitigate potential in vivo CAR-T dysfunctions, delay exhaustion, or improve CAR-T cells expansion, particularly for CAR-T compositions engineered from cells with intrinsic inferior performance. Novel CARs with T-cell activating features, such as placement of the CAR-T cells, construct downstream of the T-cell receptor promoter, resulting in a more physiologically active and less-likely exhausted phenotype [39].

Combining allo-SCT and CAR-T cells therapy is an attractive area of research to improve the outcomes further. Allo-SCT has been used in several clinical trials after CAR-T cells treatment and showed efficacy and favorable prognosis in R/R B-ALL [40,41]. Considering the high heterogeneity among and within patients, different CAR-T cell products, and continuously improved treatment strategies, consolidative allo-SCT cannot be generalized; however, it is recommended for high-risk r/r B-ALL patients with no history of allo-SCT and achieved MRD-negative CR after anti-CD19 CAR-T therapy [41]. Patients without risk factors could achieve long-term remissions with a continuous expression of the CAR in durable and functional CAR-T cells [42].

## 3. Safety Profile

With the available clinical algorithms, multidisciplinary staff must maintain competency in managing complications. Healthcare institutions must regularly review management guidelines based on current evidence, and host and product variables. Nurses play a fundamental role in educating caregivers and patients to identify and initiate early management of toxicities. Moreover, collaboration with the Department of Intensive Care is essential to tackle the most impactful complications.

A higher incidence of cytokine release syndrome (CRS) and immune cell-associated neurotoxicity syndrome (ICANS) could be associated with several factors, such as construct with a CD28 co-stimulatory domain [43], CD8^+^ CAR-T dose [44], bridging therapies in patients with the more aggressive disease [45], and the use of different grading systems to classify toxicity. The American Society for Blood and Marrow Transplantation criteria are the most objective and easy to use [46].

IL-6 released by macrophages and monocytes plays a significant role in the pathogenesis of toxicities, and extensive research efforts are focused on better understanding the mechanisms of these toxicities and defining strategies to mitigate them [47]. In addition, identifying biomarkers that are easily applicable in clinical practice will improve the treatment of post-CAR-T infusion toxicity [48].

Strategies with a risk-adapted earlier use of corticosteroids and tocilizumab to mitigate CRS are evolving [49]. The treatment of ICANS has largely been limited to supportive care and corticosteroids. More recently, some early clinical data investigated the use of Anakinra as a promising agent in the prevention and treatment of severe ICANS [50]. The investigation of adding a suicide gene that monoclonal antibodies may trigger, such as rituximab or cetuximab, or by using the CRISPR/Cas9 genome editing technology to clone a specific CAR construct to the T-cell receptor α constant (TRAC) locus resulting in uniform CAR expression and enhanced T-cell potency, needs to be considered in future CAR-T production [51]. Novel strategies are developing to interrupt these mechanisms without attenuating CAR-T efficacy. For example, the granulocyte-macrophage colony-stimulating factor (GM-CSF) is now known to promote the production of proinflammatory cytokines in CRS. Strategies to reduce GM-CSF production are being explored, such as editing out GM-CSF from effector CAR-T cells [52].

The long-term incidence of patients presenting with B-cell aplasia and, in general, patients with leukopenia, thrombocytopenia, and anemia, should not be underestimated. Therefore, future CAR constructs could target a clonally restricted B-cell marker, such as a kappa and lambda light chain of immunoglobulins, which could maintain anti-tumor activity without compromising humoral immunity, preventing B-cell aplasia [53]. Another strategy could be deleting the target antigen on normal hematopoietic stem cells, as recently shown by Kim et al. [54].

## 4. Manufacture Process

One of the future objectives must be the reduction of the production times of the CAR-T. Three to four weeks is too long for patients with R/R disease, and we highlight that 18% of patients enrolled in ELIANA could not get the product because of deterioration, death, or manufacturing failure.

Possible solutions for reducing manufacturing time include novel manufacturing processes, decentralized manufacturing, and exploring novel non-viral vector approaches of genetic engineering with a faster turnaround time [55]. For example, Jackson et al. showed that it is possible to manufacture CAR-T cells within a hospital setting using a GMP-compliant closed system [56]. As a result, the authors reduced the turnaround cell manufacturing time to eight days, lowering the cost and expediting patient treatment. Such a fast turnaround time is one of the main advantages of local manufacturing over centralized CAR-T manufacturing strategies.

Research is now pushing toward the next generation of CAR-T therapies, allogenic or off-the-shelf treatments that can be mass-manufactured from a healthy donor (HD)’s cells and used for multiple patients [57]. Off-the-shelf therapies have the potential to treat 100 patients per batch of allogeneic CAR-T, similar to a drug product, and have the advantage of being available immediately with no risk of delay or failed manufacturing. Although this technology is still being developed, sights are already set on the manufacturing efficiency and how it would compare to autologous CAR-T therapy production. HD sources include peripheral blood mononuclear cells, “universal donors” such as umbilical cord blood, induced pluripotent stem cells, embryonic stem cells, or HLA-silenced CD34+ progenitor stem cells [58]. UCART19 is a product investigated in children and adults with R/R B-ALL, with a manageable safety profile in heavily pretreated pediatric and adult patients with R/R B-cell ALL. However, allogeneic cells risk immune rejection by host T cells and allo-reactivation of the CAR-T cells via the TCR receptor against host tissues, causing graft versus host disease (GVHD) [59]. Many trials are currently employing off-the-shelf products, including a few practices with gene-edited deletion of the surface TRAC molecule to prevent GVHD [60].

## 5. Solid Tumors

Several small trials on CAR-T cells in solid tumors have been conducted; however, the research is still in its early stages. Most of the data published so far are small, preliminary results of clinical trials. No findings have shown sustained, long remissions [61]. Several factors hinder the effectiveness of cell therapy against solid tumors. First, intra-tumor heterogeneity is one where cells of a solid tumor do not all present the same mix or sufficient expression for the antigens at their surfaces; thus, CAR-T cells could spare some cancer cells that will, later on, induce a relapse [62]. An immune suppressive tumor microenvironment (TME) is the second factor. Solid tumors can interfere with proper inflammation, producing immune-suppressing agents such as the checkpoint molecule PD-L1 that will prevent immune activation. The accessibility of CAR-T cells to the tumor mass is a third critical factor. A solid group of cells stacked in layers is difficult for T cells to infiltrate. While some trials deliver the cells systemically, others aim to improve efficacy by administering CAR-T cells directly to the tumor site [63].

Several studies are underway to combine CAR-T with cytokine administration, checkpoint blockade, oncolytic viruses, radiation, and vaccines [64]. In addition, investigators have explored the use of T cells to deliver viruses into tumors directly. For example, combining CAR-T infusion with the local delivery of an oncolytic adenovirus encoding RANTES and IL-15 in preclinical models has improved homing to and the persistence of CAR-T cells at tumor sites [64].

The composition of immune cells in the tumor microenvironment is an essential element for the heterogeneity of tumors, and creates interesting yet challenging complexities when investigating dynamic interactions between cancer and immune cells [65]. Tumor transcriptomics data are informative; however, they do not immediately indicate immune cell compositions, which require computational inference. The computational algorithms are based on two categories: deconvolution approaches and gene signature [66]. Deconvolution methods define the problem as mathematical equations that model the gene expression of a tissue sample as the weighted sum of the expression profiles from the cells in the population mix. Gene signature-based approaches utilize a list of cell-type-specific gene sets. These two complementary categories of algorithms have demonstrated variable performance advantages in estimating specific immune cell types in different tumors [67]. The algorithms could help the user gain more comprehensive and robust results with CAR-T cells.

## 6. Cost

Since the first CAR-T cells therapies gained FDA approval in 2017, the one-time treatments have led to unprecedented response rates in patients with R/R lymphoid malignancies, with remarkable price tags of about $373,000 for a single infusion.

In the late 1980s, the Italian hospitals developed a calculating tariff method based on diagnosis-related groups (DRG) [68]. The DRGs have also been applied in North America [69]. DRGs are awarded by a “grouper” program based on International Classification of Diseases diagnoses, physical characteristics (gender, age), procedures, the presence of complications or comorbidities, and discharge status. By the DRG identified and the length of hospital stay, the region pays the cost of hospitalization. DRGs include all the actions necessary to treat and diagnose the patient for each treatment because patients within each category are clinically similar and are expected to employ the same level of hospital resources (fixed price).

According to DRG, CAR-T cells therapy in Italy is remunerated at $59,806 in most authorized centers. However, the actual repayment of the DRG does not correspond to the cost of the CAR-T cells procedure and, in general, of a transplant procedure [67]. Furthermore, the price does not account for manufacturing the product, managing potential long-term complications, or managing other therapy lines after relapse. The DRG model finds complex applications in this scenario, and maybe a novel model should be explicitly applied for cell therapies. For example, activity-based costing (ABC) is a tool developed to improve efficiency and control cost. The procedure is based on the concept that the production of a product or the performance of a service spends activities that consume resources [70,71]. ABC endeavors to assign costs to each of these activities, and resources so that total costs can be better understood and administrated. Finally, the pharmaceutic company introduced an outcome-based pricing model: if the treatment does not work, no one pays for it. That proposal is exciting with many problems, the least of which is that the definition of “not working” is unclear [68].

## 7. Conclusions

CAR-T cells therapy has shown unprecedented results in patients without curative options. Future work focusing on target identification, toxicity management, and manufacturing time shortening will broaden this exciting therapy’s clinical applicability and sustainability, with more prolonged remissions without additional treatment.

## Figures and Tables

**Table 1:** Main characteristics of the CAR-T constructs and clinical indications.

Name	General Description	Therapeutic Indications
**Tisagenlecleucel**	Immunocellular therapy containing tisagenlecleucel, autologous T cells genetically modified ex vivo using a lentiviral vector encoding an anti-CD19 chimeric antigen receptor.	Pediatric and young adult patients up to and including 25 years of age with B-cell acute lymphoblastic leukemia that is refractory, in relapse post-transplant, or in second or later relapse. Adult patients with R/R diffuse large B-cell lymphoma after two or more lines of systemic therapy.
**Axicabtageneciloleucel**	A CD19-directed genetically modified autologous T-cell immunotherapy. T cells are genetically modified ex vivo by retroviral transduction to express a chimeric antigen receptor comprising a murine anti-CD19 single-chain variable fragment linked to the CD28 co-stimulatory domain and CD3-zeta signaling domain.	After two or more lines of systemic therapy, adult patients with R/R diffuse large B-cell lymphoma and primary mediastinal large B-cell lymphoma.
**Lisocabtagenemaraleucel**	Anti-CD19 single-chain variable fragment (scFv) targeting domain for antigen specificity, a transmembrane domain, a 4-1BB costimulatory domain hypothesized to increase T-cell proliferation and persistence, and a CD3-zeta T-cell activation domain.	After two or more lines of systemic therapy, adult patients with R/R large B-cell lymphoma, including diffuse large B-cell lymphoma, not otherwise specified, high-grade B-cell lymphoma, primary mediastinal large B-cell lymphoma, and follicular lymphoma grade 3B.
**Brexucabtageneautoleucel**	Autologous peripheral blood T-lymphocytes (PBTL) that have been transduced with a retroviral vector expressing a chimeric antigen receptor (CAR) consisting of an anti-CD19 single-chain variable fragment (scFv) coupled to the zeta chain of the T-cell receptor (TCR)/CD3 complex (CD3 zeta) and the costimulatory signaling domain CD28.	Treatment of adult patients with R/R mantle cell lymphoma.
**Idecabtagenevicleucel**	Anti B-Cell maturation antigen (BCMA) scFv fused to the CD137 (4-1BB) co-stimulatory and CD3ζ signaling domains.	Adult patients with R/R multiple myeloma after four or more prior lines of therapy, including an immunomodulatory agent, a proteasome inhibitor, and an anti-CD38 monoclonal antibody.
**Ciltacabtageneautoleucel**	BCMA-targeted T-cell therapies are directed against two BCMA epitopes (VH1 and VH2) to confer improved affinity for BCMA-expressing cells.	Not authorized. Trials ongoing in R/R multiple myeloma to both immunomodulatory agents and proteasome inhibitors, or with at least three prior lines of therapy and previously exposed to anti-CD38 monoclonal antibody.

## Data Availability

Data sharing is not applicable. No new data were created or analyzed in this study.
